# Differential effects of antibiotics in combination with G-CSF on survival and polymorphonuclear granulocyte cell functions in septic rats

**DOI:** 10.1186/1471-2334-8-55

**Published:** 2008-04-30

**Authors:** Artur Bauhofer, Markus Huttel, Wilfried Lorenz, Daniel I Sessler, Alexander Torossian

**Affiliations:** 1 Institute of Theoretical Surgery, Philipps-University Marburg, Baldingerstrasse, 35033 Marburg, Germany; 2 Department of Outcomes Research, The Cleveland Clinic, Cleveland, OH 44195, USA; 3 Clinic of Anesthesiology and Critical Care, Philipps-University Marburg, Baldingerstrasse, 35033 Marburg, Germany; 4 MEDA Pharma GmbH & Co KG, Benzstrasse 1, 61352 Bad Homburg, Germany

## Abstract

**Background:**

In addition to their antimicrobial activity, antibiotics modulate cellular host defence. Granulocyte-colony stimulating factor (G-CSF) is also a well known immunomodulator; however little is known about the interactions of G-CSF with antibiotics. We investigated in septic rats the effects of two antibiotic combinations with G-CSF.

**Methods:**

In two clinic modelling randomised trials (CMRTs), male Wistar rats were anesthetized, given antibiotic prophylaxis, had a laparotomy with peritoneal contamination and infection (PCI), and were randomly assigned (n = 18 rats/group) to: 1) PCI only; 2) PCI+antibiotic; and, 3) PCI+antibiotic+G-CSF prophylaxis (20 μg/kg, three times). This sequence was conducted first with 10 mg/kg coamoxiclav, and then with ceftriaxone/metronidazole (Cef/met, 10/3 mg/kg). In additional animals, the blood cell count, migration and superoxide production of PMNs, systemic TNF-α and liver cytokine mRNA expression levels were determined.

**Results:**

Only the combination coamoxiclav plus G-CSF improved the survival rate (82 vs. 44%, p < 0.001). Improved survival with this combination was accompanied by normalised antimicrobial PMN migratory activity and superoxide production, along with normalised systemic TNF-α levels and a reduced expression of TNF-α and IL-1 in the liver.

**Conclusion:**

There are substantial differences in the interaction of antibiotics with G-CSF. Therefore, the selection of the antibiotic for combination with G-CSF in sepsis treatment should be guided not only by the bacteria to be eliminated, but also by the effects on antimicrobial functions of PMNs and the cytokine response.

## Background

Source control and antibiotic treatment are the mainstays in the treatment of infectious disease. Interestingly, antibiotics not only directly reduce bacterial growth; they also modulate cellular host defence, especially the antimicrobial functions of monocytes and granulocytes [1]. Some antibiotics enhance cellular host defence mechanisms, but others have adverse effects. How these alterations occur remains poorly understood, but cytokines and chemokines are throught to be important regulators of antimicrobial functions [2].

Granulocyte colony-stimulating factor (G-CSF) is a glycopeptide which stimulates the granulocyte lineage and stem cells. G-CSF also suppresses release of pro-inflammatory cytokines such as TNF-α by macrophages [3]. G-CSF stimulates neutrophil recruitment and chemotaxis [4], increases phagocytosis of bacteria and production of free radicals [5], and retards normal granulocyte apoptosis [6]. The beneficial effects of G-CSF have been demonstrated in different animal models of sepsis [7-12]. In clinical trials, recombinant G-CSF (filgrastim) proved to be effective in diabetic foot ulcers [13] and high-risk febrile neutropenia [14], but not for community acquired pneumonia [15].

To evaluate the interaction of different antibiotics with G-CSF, we used clinic modelling randomised trials (CMRTs). The inclusion of an i.v. antibiotic prophylaxis is one important feature of CMRTs for modeling clinical complexity. Others include appropriate use of anaesthesia, volume loading, laparotomy, peritoneal contamination and infection with human stool bacteria (PCI), suitable postoperative analgesia, and complicating risk factors such as hypothermia and blood loss which we have assessed previously [11,16]. Furthermore it was validated and confirmed extensively by our group in terms of microbiologic characterization and reproducibility, as shown by dose-mortality relationship [17]. In addition characteristics of randomised clinical trials such as sample size calculation, randomization, double-blind design, analysis of morbidity, intent to treat analysis and adequate statistics are included in CMRTs. CMRT characteristics are summarised in a Table [see Additional file 1].

Using this model, we have evaluated cephalosporins, aminoglycosides, fluoro-quinolones, and carbapenemes in combination with G-CSF. All antibiotics are routinely used for clinical peritonitis treatment at our institution. Interestingly, 7 antibiotic/G-CSF combinations improved survival, but 4 others did not [12,17]. For combination, one of the best antibiotics (coamoxiclav) and one of the worst antibiotics (Cef/met) with regard to survival were selected for further investigations analyzing PMN function and pro-inflammatory organ cytokine expression and systemic concentrations. We hypothesised that positive antibiotic/G-CSF interactions resulting in a reduced mortality rate after sepsis depend on improved PMN function and reduced pro-inflammatory organ cytokine expression and reduced systemic cytokine levels.

## Methods

The study was performed with permission of the animal welfare committee in Giessen, Hessen, Germany. 179 male Wistar rats, 220–280 g (Charles River Wiga, Sulzfeld, Germany) were used. They were given standard diet (Altromin, Lage, Germany) and water ad libitum.

### Protocol

Two independent trials were performed to analyse survival. Separate animals were used for cytokine and PMN function analyses. At arrival, animals were assigned to study groups by simple random permutation using ear marks to: 1) peritoneal contamination and infection (PCI) only; 2) PCI + antibiotic prophylaxis, and 3) PCI + antibiotic + G-CSF prophylaxis (n = 18 rats/group). In the first trial, 10 mg/kg coamoxiclav was given; and in the second trial, ceftriaxone/metronidazole (Cef/met, 5/2 mg/kg) was given. Liver samples for mRNA analysis were taken from additional animals (3 rats/group) from all six treatment groups. Blood cell parameters and PMN functions were assessed in main group animals and in addition in the groups G-CSF only and PCI only (n = 9 rats/group).

The rats were deprived of food 12 hours before surgery. Appropriate animals received 20 μg/kg G-CSF (Filgrastim, Amgen, Munich, Germany) or placebo (Ringer's solution) as a subcutaneous injection at three times: 12 hours before surgery and 12 and 36 hours after surgery (PCI). This dose regimen was most effective as determined in dose response curves with G-CSF in the rat PCI model [12,17]. The other groups were given equal volumes of placebo (Ringer's solution).

One hour before surgery, the animals were anaesthetised with 0.08 mg/kg fentanyl and 4 mg/kg droperidol, given intraperitoneally. In the analgo-sedated, spontaneously breathing animals a tail vein was cannulated. Animals received intravenous antibiotic prophylaxis (one hour before and one hour after surgery) with coamoxiclav (Glaxo Smith Kline, Munich, Germany), or with ceftriaxone (Roche, Grenzach-Wylen, Germany) combined with metronidazole (Serag-Wiessner, Naila, Germany).

Using an antiseptic technique, a 2-cm-long midline incision was performed and 1.5 ml/kg standardized human stool inoculum (diluted 1:2.5 in Ringer's solution) was injected into the pelvic region. The wound was closed in two layers using an interrupted vicryl 3-0 suture.

Postoperative analgesia consisted of 20 mg/kg tramadol (Mundipharm, Limburg, Germany) injected subcutaneously once daily. Postoperatively, *ad libitum *food and water was provided. The animals were checked carefully at least twice daily for mortality and clinical signs. When an animal appeared to be weak, it was kept separately from the others to prevent cannibalism. Animals surviving throughout the 120-hour observation period were sacrificed by inhalation of CO_2_.

### Blood cell parameters

Under fentanyl 0.08 mg/kg and droperidol 4 mg/kg narcosis, 2000 I.E. heparin (Liquemin, Roche AG, Basel, Switzerland) was given intravenously. Blood was withdrawn by puncture of the retro-orbital venous plexus. The white blood cell count (WBC) was determined in an automated counter optimized for rat blood (Coulter Max-M, Krefeld, Germany). Granulocytes were purified on a discontinuous histopaque 1119/1077 gradient for 30 minutes at 700 × g at room temperature. The cells were washed with PBS buffer on ice and contaminating erythrocytes were lysed with distilled water for 30 seconds. Viability, purity, and the cell number were determined by trypan-blue staining and counting in a Neubauer chamber.

### PMN antimicrobial functions

Migration of PMNs was analyzed in Boyden chambers as described by Cates at al. [18]. Zymosan-activated fetal calf serum (10%) was the stimulant. The number of PMNs adhering to the filter on the side orientated towards the chemoattractant was counted in 10 randomly selected high-power fields (10 × ocular and 40 × objective). To determine the chemotactic activity in the wells with stimuli, the basal migration of PMNs from untreated animals without stimuli was set to 100%.

The production of superoxide anions was analyzed in a cytochrome-C microplate assay using a modified method from Mayo et al. [19]. Each test was performed in triplicate. For stimulation the cells were incubated with 2 μM PMA (phorbol 12-myristate 13-acetate). The nonspecific O_2_^- ^production was determined by adding 10 μM superoxide dismutase.

### TNF-α plasma levels

EDTA anti-coagulated blood was immediately centrifuged and plasma was stored at -70°C until assayed. Cytokine TNF-α concentration was quantified with rat-specific ELISA kits (Biosource, Camarillo, CA, USA).

### Cytokine mRNA expression

Rats were sacrificed with a lethal dose of fentanyl and droperidol 24 hours after PCI. Pieces of excised liver weighing about 100 mg were frozen in liquid nitrogen and then stored at -70°C. RNA was extracted with RNA-Clean kits (ASG, Heidelberg, Germany). Briefly, extracted RNA was stabilized with 40 U/μl RNasin^® ^(Promega, Madison, WI, USA). For semi-quantitative, competitive RT-PCR, a multi-specific competitor fragment for rat cytokines with the conditions published by Siegling et al. [20] was used.

Before amplification of the cytokine TNF-α, the housekeeping gene β-actin was amplified to check the efficiency of the reverse transcriptase reaction. When needed a correction was included in order to start with the same amount of c-DNA in each tube (cDNA equivalent of about 5 ng total RNA). The competitor fragment was diluted 1:4 in 3 dilution steps starting with 2 pg; if needed, a further dilution step was included. After PCR the samples were separated in a 1% agarose gel, stained with ethidium bromide, digitalized and analyzed with the Gelscan Software (BioSciTec, Marburg, Germany). For quantification, only bands with similar intensity on the grey scale between the competitor fragment and the cytokines were used.

### Statistical analysis

Survival rates were analysed with the Chi-square test and survival curves with the log rank tests. Cellular and cytokine data were assessed by ANOVA analysis of variance. If significant results were obtained in the global test, *post hoc *tests were performed with a Bonferroni correction. All tests were performed using the SPSS statistic software package [21]. Cytokine and functional data are presented as means ± SEMs. P < 0.05 was considered statistically significant.

## Results

### Mortality

**Figure 1:**
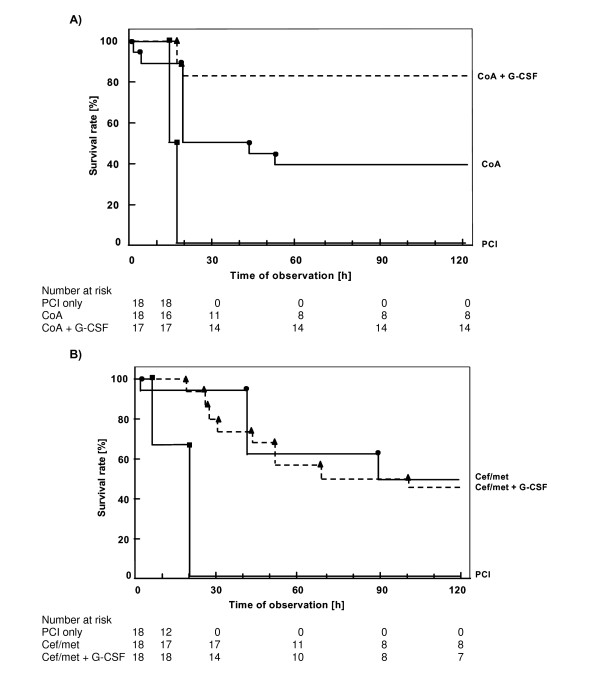
**Kaplan-Meier 120 h survival analysis of septic rats from two independent CMRTs**. Each group included n = 18 rats. A) Rats with coamoxiclav prophylaxis (10 mg/kg), coamoxiclav plus G-CSF prophylaxis before PCI and PCI without antibiotic prophylaxis. Significant differences were found by the Log-Rank Test over all groups (p < 0.001, df = 2) and in paired comparison PCI only versus coamoxiclav and PCI versus coamoxiclav + G-CSF (p < 0.001), coamoxiclav versus coamoxiclav plus G-CSF (p < 0.05) in the intent to treat analysis. B) Rats with ceftriaxone/metronidazole (Cef/met) prophylaxis (5/2 mg/kg), Cef/met plus G-CSF prophylaxis before PCI and PCI without antibiotic prophylaxis.

One rat died during anaesthesia, but before PCI, in the first trial in the G-CSF group; all other rats were included into the analysis. There were no further complications related to the perioperative injections or surgery. No rat survived without antibiotic prophylaxis. Coamoxiclav prophylaxis increased survival to 44% (8/18) and survivial further increased to 82% (14/17) when prophylactic G-CSF was included (p < 0.001, Figure 1A).

In the Cef/met trial, survival of the antibiotic prophylaxis group was 44% (8/18) which was nearly identical to the 39% (7/18) survival when Cef/met was combined with G-CSF (Figure 1B).

### Blood cell parameters

In rats without PCI, G-CSF administration doubled the WBC to 22.7 ± 2.0 × 10^6 ^cells/ml and increased the PMN fraction to 31 ± 3.5% (Table 1). The systemic white blood cell count decreased and the PMN fraction increased 24 hours after PCI. In infected rats, there was no significant changes in WBC count or PMN fraction with either antibiotic prophylaxis (coamoxiclav or Cef/met) alone or in combination with G-CSF.

**Table 1:** Blood cell parameters

	**Control**	**G-CSF**	**PCI**	**coamoxiclav**	**coamoxiclav + G-CSF**	**Cef/met**	**Cef/met + G-CSF**
**WBC (10**^6 ^**cells/ml)**	11.7 ± 0.9	22.7 ± 2.0^#^	8.0 ± 0.7	8.3 ± 0.6	9.6 ± 0.8	8.9 ± 0.7	10.7 ± 2.1
**PMN (%)**	16 ± 1.6	31 ± 3.5*	34 ± 3.5^#^	30 ± 4.6^§^	26 ± 3.7	37 ± 2.3^#^	35 ± 1.9^#^

Blood cell parameters from naive and septic rats. Naive, non-infected control rats, naive rats treated with G-CSF only (20 μg/kg, 12 h before and 12 h after PCI), PCI rats without prophylaxis, PCI rats with antibiotic prophylaxis (coamoxiclav = CoA or ceftriaxone/metronidazole = Cef/met) and PCI rats with antibiotic plus G-CSF prophylaxis were analysed. Blood was sampled 24 h after PCI. Data are presented as means ± SEM from n = 9 animals/group. ANOVA analysis of variance revealed P < 0.001, df = 6 and post hoc tests of with Bonferroni correction obtained ^#^P < 0.001 versus control, *P < 0.01 versus control, ^§^P < 0.05 versus control.

### PMN antimicrobial functions

**Figure 2:**
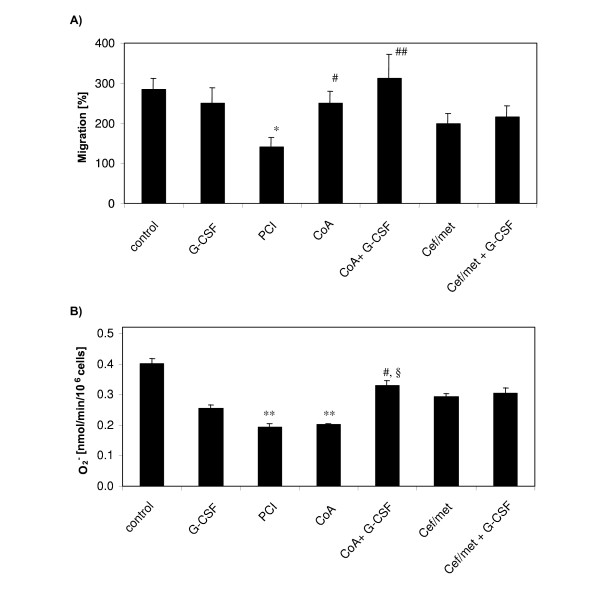
**PMN antimicrobial functions from control (non-infected) and septic rats**. Naive, non-infected control rats, naive rats treated with G-CSF only (20 μg/kg, 12 h before and 12 h after PCI), PCI rats without prophylaxis, PCI rats with antibiotic prophylaxis (coamoxiclav = CoA or ceftriaxone/metronidazole = Cef/met) and PCI rats with antibiotic plus G-CSF prophylaxis were analysed. Blood was sampled at 24 h after PCI from n = 9 animals each condition. Migration was assessed with a Boyden camber test. Granulocyte superoxid anion formation was determined with a cytochrom-C microplate assay. Data are presented as mean ± SEM. ANOVA analysis of variance revealed for migration P < 0.05 and for superoxide anion formation P < 0.001, df = 6 and post hoc tests of with Bonferroni correction obtained *P < 0.01 versus control, **P < 0.001 versus control, ^#^P < 0.05 versus PCI, ^##^P < 0.01 versus PCI, ^§^P < 0.05 versus coamoxiclav.

PMN purity was comparable in each cell preparation. PMN antimicrobial functions, specifically migration and superoxide anion formation, were depressed after PCI (Figure 2). Compared to PCI alone, coamoxiclav prophylaxis (P < 0.05) and in combination with G-CSF (P < 0.01) improved the migratory activity of PMNs to approximately the level of cells from naive animals. In contrast, Cef/met alone or in combination with G-CSF did not significantly improve PMN migration.

Superoxide anion formation was also depressed by PCI. With each antibiotic alone, no significant restoration of the superoxide production was observed. Only the combination coamoxiclav and G-CSF increased superoxide production capacity significantly compared to PCI alone (P < 0.01).

### Cytokine levels

**Figure 3:**
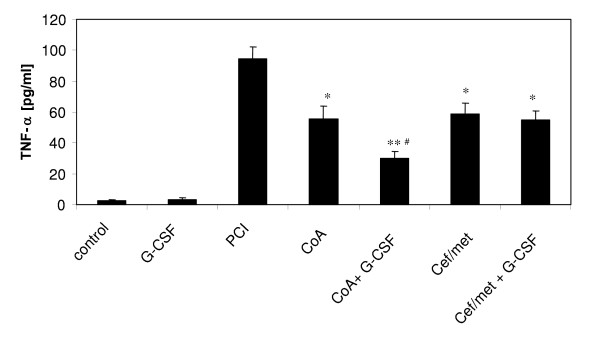
**TNF-α protein levels in the plasma 24 hours after PCI**. Naive, non-infected control rats, naive rats treated with G-CSF only (20 μg/kg, 12 h before and 12 h after PCI), PCI rats without prophylaxis, PCI rats with antibiotic prophylaxis (coamoxiclav = CoA or ceftriaxone/metronidazole = Cef/met) and PCI rats with antibiotic plus G-CSF prophylaxis were analysed. Cytokine levels were determined by ELISA. ANOVA analysis of variance revealed P < 0.001 for n = 9/group and post hoc tests of with Bonferroni correction obtained a significant increase of the cytokine level in all infected animals compared to control (P < 0.01) and *P < 0.05 versus PCI, **P < 0.01 versus PCI and ^#^P < 0.05 versus coamoxiclav. Data are presented as mean ± SEM.

Systemic protein levels of TNF-α were significantly increased 24 hours after PCI in all infected groups (Figure 3). TNF-α plasma levels were already decreased compared to PCI by an antibiotic prophylaxis with coamoxiclav or with Cef/met (p < 0.05), whereby a further reduction was obtained by the addition of G-CSF only in the coamoxiclav group (p < 0.05 versus coamoxiclav).

**Figure 4:**
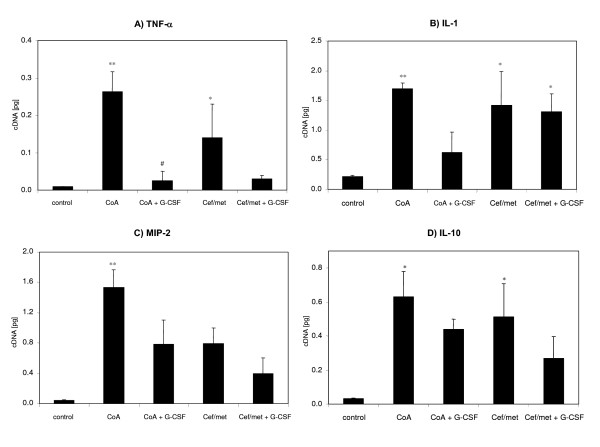
**Cytokine mRNA expression profiles from control and septic rats**. Rats were 24 hours after PCI sacrificed, and liver probes were analyzed by reverse, semi-quantitative, competitive RT-PCR. Results are shown for the cytokines TNF-α, IL-6, IL-1, MIP-2 and IL-10. The data are expressed as means + SEM (n = 3 animals each condition). ANOVA analysis of variance revealed for the different cytokines P < 0.001, df = 4 and post hoc tests of with Bonferroni correction obtained *P < 0.05 versus control, **P < 0.01 versus control, ^#^P < 0.05 versus coamoxiclav.

In liver biopsies, mRNA expression of TNF-α, IL-1, MIP-2 and IL-10 were elevated in the antibiotic prophylaxis groups compared to uninfected controls with the exception MIP-2 after antibiotic prophylaxis (Figure 4). TNF-α was further significantly reduced (P < 0.05) by the combination coamoxiclav plus G-CSF and tended to be reduced by the prophylaxis Cef/met plus G-CSF (P = 0.11) (Figure 4A). A further reduction of the cytokine IL-1 was also observed after prophylaxis with coamoxiclav plus G-CSF but not in combination with the other antibiotic (Figure 4B). The MIP-2 and IL-10 levels were in combination with both antibiotics plus G-CSF not different from uninfected controls (Figure 4C and 4D).

## Discussion

The cytokine G-CSF is a glycopeptide that antagonizes the neutrophil depression associated with bone marrow transplantation or sepsis [22,23]. Cef/met alone reduces the mortality rate in rats with peritoneal contamination and infection, but no additive effect was obtained by combining it with G-CSF. Only the combination coamoxiclav plus G-CSF was highly effective. This result is consistent with a previous report [24].

No differences were observed in the white blood cell count or PMN ratio after G-CSF prophylaxis in combination with the two antibiotics. The absolute number of the white blood cells thus does not appear to be a crucial factor explaining the efficacy of various antibiotics in combination with G-CSF.

Antimicrobial PMN functions, namely the migratory activity and superoxide production are differently regulated. In obstetric patients without antibiotic treatment reduced PMN migration was predictive for infections in cases of preterm premature rupture of membranes [25]. From the literature it is known that G-CSF stimulates cellular immune defence mechanisms and alters the cytokine network. G-CSF induces the granulocyte recruitment and enhances their function at the site of infection, e.g. peritoneally. Especially the production of superoxide anions could be normalized in septic animals by G-CSF treatment [26], but these results did not explain the antibiotic differences.

Unfortunately, only very few results are available assessing the antimicrobial activity and interaction of G-CSF with different antibiotics. Beneficial effects of G-CSF in combination with fluoro-quinolones such as ofloxacin on the antimicrobial functions were reported [27]. With the antibiotics alone several investigations were performed. Coamoxiclav increases the phagocytotic activity of PMNs in vitro tested with un-opsonized and opsonized Staphylococcus aureus [28]. The super oxide formation was unchanged in this trial. Only very high coamoxiclav concentrations (100 mg/l) modestly suppress this function [28]. In contrast, for ceftriaxone it was shown that the PMN migration induced by the peptide FMLP (formyl-methionyl-leucyl-phenylalanine) was suppressed [29]. This was also the case for the phagocytosis of adherent PMNs but not for PMNs in solution [30]. The respiratory burst was unchanged in this group. In vitro metrodinazole seems to have no influence to the migratory activity, phagocytosis and the respiratory burst [31,32]. Thus although ceftriaxone is a potent antibiotic, its effects on PMN antimicrobial functions are less impressive. The difference in interaction of G-CSF with the two antibiotics most probably results from differences in phagocyte activity, although a direct influence on the antibiotic activity can not be ruled out.

There is a close interaction of the antimicrobial PMN functions and the cytokine cascade activated during infections. In our trial, only the combination of G-CSF and coamoxiclav decreased systemic TNF-α. In the liver, the cytokine mRNA expression showed a heterogeneous picture. TNF-α was also significantly reduced by the combination coamoxiclav plus G-CSF compared to antibiotic only. This was not the case with the Cef/met, but in the group without G-CSF prophylaxis, a lower expression of TNF-α was observed after Cef/met prophylaxis. IL-1 mRNA levels were not different from control after prophylaxis with coamoxiclav plus G-CSF, but after prophylaxis with Cef/met plus G-CSF they remained still elevated. The combinations showed no difference between the two antibiotics regarding the cytokines MIP-2 (macrophage inflammatory protein-2) and IL-10. MIP-2 is a chemokine predominantly produced by mononuclear leucocytes which attracts PMNs and IL-10 is a well known anti-inflammatory cytokine. Surprisingly these two factors seem to be less important for the different antibiotic effect in combination with G-CSF, at least as measured in the liver. We can not exclude that they may play an important role in other organs or systemically.

Antibiotics have different effects on Cytokines. A recent *in vitro *study demonstrated that coamoxiclav reduces mRNA expression of pro-inflammatory cytokines such as TNF-α, IL-1, IL-6 from PMNs stimulated with Klebsiella pneumoniae; in contrast, expression of the anti-inflammatory cytokine IL-10 did not increase [1]. *In vitro *work also demonstrates that TNF-α release is increased by ceftriaxone, whereas other cytokines including IL-1, IL-6, and IL-8 were unchanged [33,34]. TNF-α is able to enhance the undirected migration and to prime PMNs for a second stimulus [35]. Grutoski et al showed that the effect of TNF-α is much more complex, since TNF-α induces a factor which suppresses migration and antimicrobial activity of PMNs in a paracrine manner [36]. Elevated TNF-α levels are regarded to be adverse for the antimicrobial functions of PMNs and consequently for the outcome from major infections such as sepsis. In contrast G-CSF has anti-inflammatory properties [37] and the potential to enhance migration and the oxidative burst in septic patients [38]. The antibiotic differences have also implications for the possible clinical use of G-CSF. In a recent randomised clinical trial with a G-CSF prophylaxis for improvement of the outcome of high risk patients after colorectal cancer surgery also two different antibiotics (cefuroxime/metronidazole and ofloxacin/metronidazole) were used [39]. In this trial again one antibiotic (cefuroxime/metronidazole) in combination with G-CSF was more effective to reduce the complication rate and length of hospital stay and to improve quality of life. The effects from cytokines and PMN functions are differentially regulated by antibiotics.

## Conclusion

There are substantial differences in the interaction of antibiotics with G-CSF. G-CSF was only able to improve survival in combination with coamoxiclav. Increased survival may be explained by an improvement of antimicrobial PMN functions and by an altered cytokine expression which regulate these functions. Therefore, the selection of the antibiotic for combination with G-CSF in sepsis treatment should be guided not only by the bacteria to be eliminated, but also by the effects on antimicrobial functions of PMNs and the cytokine response. The antimicrobial activity will be optimised by a positive combination of direct antibiotic effects and indirect effects of the innate immune system like the PMNs.

## List of abbreviations

AB: antibiotic; Cef/met: ceftriaxone/metrodinazole; CMRT: clinic modelling randomized trial; FMLP: formyl-methionyl-leucyl-phenylalanine; G-CSF: granulocyte-colony stimulating factor; IL: interleukin; MIP: macrophage inflammatory protein 2; PCI: peritoneal contamination and infection; PMN: polymorph nuclear granulocyte; TNF-α: tumour necrosis factor alpha; WBC: white blood cell count.

## Declaration of competing interests

The authors declare that they have no competing interest. The work was in part supported by a grant of the Deutsche Forschungsgemeinschaft (DFG).

## Authors' contributions

AB designed the study, supervised the experiments and wrote the manuscript in most parts. MH performed the experiments in most parts. DS helped to improve the manuscript. WL had the study idea and gave input to the study design and experimental procedure. AT was involved in data analysis, experimentation and writing of the manuscript

## Pre-publication history

The pre-publication history for this paper can be accessed here:



## Supplementary Material

Additional file 1 Characteristics of clinic modelling randomized trials (CMRTs) Click here for file
