# Supplementary material for: Differential effects of antibiotics in combination with G-CSF on survival and polymorphonuclear granulocyte cell functions in septic rats
Source: BMC Infect Dis. 2008 Apr 30;8:55. doi: 10.1186/1471-2334-8-55 (PMC2386131; doi:10.1186/1471-2334-8-55)
Supplement: Additional file 1 — Characteristics of clinic modelling randomized trials (CMRTs) [file 1471-2334-8-55-S1.doc]

**Clinic modeling randomized trials (CMRTs): *rationale and characteristics***

- Modeling clinical trials (scenario and methodology), before or after conducting a clinical trial
- Modeling clinical complexity is more important than species differences
- Modeling treatment effects as expected or warranted in the clinical scenario (δ, α and β): e.g. high sample size
- Searching for positive and negative results

| **Modeling the clinical situation:** | **Modeling randomized trials:** |
| --- | --- |
| - Developed with theoretical surgeon | - Sample size calculation (δ = 0.3, 2α = 0.05, 1-β = 0.9 |
| - Adequate anesthesia (fentanyl/droperidol) | - Randomized allocation to the groups |
| - Preoperative antibiotic prophylaxis | - Double-blind design |
| - Perioperative volume substitution | - Clinically relevant endpoint (five day mortality) |
| - Operation (laparotomy) | - Evaluation of morbidity |
| - Peritoneal contamination with human stool | - Search for positive and negative results |
| - Outcome adapted to the clinic: high mortality rate | - “intent to treat” rule |
| - Postoperative analgesia | - Adequate statistical analysis |
